# Association between oral health-related behaviors and quality of life of adolescents among three provinces in Northwest China

**DOI:** 10.3389/fpubh.2024.1407623

**Published:** 2024-11-25

**Authors:** Xiao Hu, Can Wang, Jianghong Gao, Jiangang Tian, Lingling Li, Zhige Li, Kaixin Guo, Ruizhe Huang

**Affiliations:** ^1^Key Laboratory of Shaanxi Province for Craniofacial Precision Medicine Research, College of Stomatology, Xi'an Jiaotong University, Xi'an, Shaanxi, China; ^2^Yinchuan Stomatological Hospital, Yinchuan, Ningxia Hui Autonomous Region, Ningxia, China; ^3^College of Stomatology, Lanzhou University, Lanzhou, Gansu, China

**Keywords:** OHRQoL, adolescents, different provinces, Northwest China, oral health-related behaviors

## Abstract

**Objective:**

To assess the oral health-related quality of life (OHRQoL) of adolescents in Northwest China, and to explore the relationship between sociodemographic characteristics, oral health-related behaviors and OHRQoL.

**Methods:**

A cross-sectional survey of adolescents aged 12–15 years in Shaanxi, Gansu province and Ningxia Hui Autonomous Region of Northwest China was conducted by stratified cluster random sampling. Oral examinations were performed with World Health Organization (WHO) standards, and the condition of crowns and periodontal was recorded. Adolescents' oral health-related behaviors and OHRQoL were collected by questionnaire. Chi-square test and binary logistic regression analysis were used to examine the relationship.

**Results:**

A total of 7,648 subjects were included. The prevalence of low OHRQoL which defined as a score higher than 0, was 83.8%. A low OHRQoL was most prevalent in Gansu Province and least prevalent in Shaanxi Province. Younger (OR = 0.73, CI = 0.60–0.87), female (OR = 1.27, CI = 1.11–1.44), rural (OR = 1.44, CI = 1.21–1.72), and mother with less education (OR = 0.69, CI = 0.60–0.81) all showed statistically significant influence on teenagers' OHRQoL. From a behavior perspective, teens' OHRQoL was correlated with their frequency of sugar consumption (OR = 1.72, CI = 1.41–2.10), dental appointment rate (OR = 1.29, CI = 1.064–1.57), self-assessment of oral health (OR = 3.09, CI = 2.29–4.19), DMFT index (OR = 1.19, CI = 1.04–1.37), number of teeth calculus (OR = 1.20, CI = 1.01–1.43), and dental trauma (OR = 0.47, CI = 0.39–0.57) over the previous year; however, brushing their teeth was not statistically significant.

**Conclusions:**

Generally speaking, oral health-related behaviors—such as eating more sugary foods and scheduling dental appointments within the last year—have a significant negative impact on the quality of life of adolescents in Northwest China. Female, and adolescents from families with low educational backgrounds are more likely to be affected by such behaviors in their daily lives.

## Background

Oral health is the foundation of whole-body health, and poor oral conditions can have a negative impact on a person's quality of life. Adolescents are in a period of rapid growth, and oral status will not only affect oral function, aesthetics and social interaction, but also have an impact on growth and development.

Traditional clinical measures do not adequately reflect the impact of oral health on personal, social, and mental health. Considerable researches are paying attention to Machine Learning algorithms to screen dental caries, this kind of assessment still need further studies to refine its items (1, 2). Oral health-related quality of life (OHRQoL) refers to a person's subjective assessment of their oral status, function, social-emotional, wellbeing, and satisfaction, reflecting the impact of oral disease on physical, psychological, and social satisfaction in daily life. Studies have shown that oral condition is the most direct factor affecting OHRQoL, and various factors such as sociodemographic characteristics and oral health behaviors are also related to it (3). M Aarts's study show that older and female children with oligodontia aged 8–29 years old scored lower on appearance of the face, appearance distress, social function, and psychological function (4). A study focus on Brazilian adolescents (5) prove that the peers undergoing orthodontic treatment had better quality of life on functional limitations, emotional wellbeing, social wellbeing which is consistent with the other studies (6, 7).

China has a wide geographical area, and the economic development of the north and south regions is unbalanced. Northwest China's geographical environment is relatively complex and its economy is at the middle or lower reaches of the country. It is one of the seven geographical regions and one of the concentrated areas of ethnic minorities in China. Factors such as geographical and cultural differences are playing significant roles. With profound historical and cultural heritage, it's an important channel and node of the Silk Road Economic Belt. Affected by natural geography, human and historical environment, the social and economic development level of the five provinces in northwest China is relatively backward (8), and the diet structure is relatively complex (9).

The oral condition of adolescents in northwest China is poor, studies have shown that the prevalence of caries and periodontal status of adolescents in Northwest China is significantly worse than that in East China (10–12). Oral health-related behaviors, mainly including tooth brushing, sugar ingestion and other nutritional levels, frequency of dental appointment, among others, can affect the occurrence of oral diseases. Moreover, literacy levels, cultural and environmental factors are social determinants of health engaged also with oral health (13, 14). Being so, it is meaningful to pay attention to the oral problems of adolescents in Northwest China and improving the quality of daily life of these individuals. The purpose of this study was to assess the OHRQoL of adolescents aged 12–15 years in Northwest China, and to explore the relationship between socio-demographic, oral health behaviors, and OHRQoL.

## Materials and methods

### Survey object

This observational cross-sectional study was conducted according to the oral health epidemiological investigation protocol recommended by WHO. The survey was conducted among 12–15 years adolescents in three provinces of Northwest China (15). The sample size was estimated as follows: *N*=t^2PQ/d^. Among them, P was the prevalence rate of caries in 12-year-old children in the fourth national oral epidemiological survey, *P* = 34.5%; Q = 1-P; Significance level α = 0.05, t = 1.96; Deviation d = 0.1P; The control loss to follow-up rate was <10%, expand the sample size by 3 times, and the final included sample size was higher than the calculated minimum sample size.

A multi-stage stratified cluster random sampling method and questionnaire survey was used to conduct oral examination and collect information among 12–15 years old adolescents in Shaanxi Province, Gansu Province and Ningxia Hui Autonomous Region. All examiners have gone through standardized training and standard conformance test, and Kappa value are >0.8. We randomly selected 2 districts and 2 counties from each province, and then 3 secondary schools were selected from each county or district at random. Finally, all subjects in each secondary school would be collected in our study. This study was approved by the Ethics Committee of the Chinese Stomatological Association (Approval No. 2014-003).

### Oral examination

Oral examination was performed by a dental practitioner with qualified experience by World Health Organization (WHO) standards. The prevalence of caries was assessed by the number of decayed, missing and filled teeth (DMFT index). The periodontal status was measured by the number of teeth with gingival bleeding and calculus.

### Questionnaire survey

The Fourth National Oral Epidemiology Questionnaire, which was utilized in earlier researches on Chinese adolescents (11, 16), was adopted by our study. Both adolescents and their parents were informed about the purpose of the study. All participants gave their informed consent, and they were allowed as much time as required to finish filling out the survey. The main aspects are as follows: (1) social demographic: age, gender, household registration, parental education; (2) Oral health related behaviors: frequency of brushing teeth every day, frequency of sugary food intake, and dental appointment in the past 1 year; (3) Oral health status: Self-evaluate oral health and general health status.

### Oral health related quality of life

Adolescents' oral health issues are widespread and likely to have a negative influence on quality of life, hence it is vital to have a single standardized self-report health status measure for them. The Child version of oral impact on daily performance (Child-OIDP) scale is an age-specific questionnaires measuring 12–15 years old childrens' oral health outcomes. It assessed the impact of oral problems on eight everyday behaviors: eating, pronunciation, brushing or gargling, schooling, sleeping, smiling, emotion and socializing. The questions were worded as follows: “How much does the condition of your oral health affect your eating?” Each question contained five options, which were severe impact, moderate impact, slight impact, no impact, and unclear. Questionnaires with incomplete answers or the option “unclear” were excluded. The total score was the sum of the eight question scores, and the final impact score was the total score divided by 24 and multiplied by 100 (17). Those who received a score of more than zero on any given question were considered to be affected by oral disease. The total prevalence of OHRQoL was determined using the information from all eight questions. The higher the total score, the worse the quality of life related to oral health.

### Statistical analysis

Statistical analysis was performed using IBM SPSS Statistics v 26.0. Descriptive analysis was performed for all data, mean standard deviation was calculated for quantitative data, and count data was expressed as rate or percentage. With the prevalence of OHRQoL and Child-OIDP score as dependent variables, Chi-square test, Mann-Whitney test and Kruskal-Wallis test were used for univariate analysis because the samples did not conform to the normal distribution. Independent variables with *P* < 0.05 in univariate analysis were included in multivariate analysis, and the relationship between independent variables and the prevalence of OHRQoL was analyzed by binary logistic regression model. *P* < 0.05 was considered statistically significant.

## Results

A total of 7,648 adolescents were included in the analysis. Among them, Shaanxi Province, Gansu Province and Ningxia Hui Autonomous Region included 1,666 (21.8%), 2,987 (39.1%), and 2,995 (39.2%), respectively. Girls account for 50.6%, rural accounts for 84.7%, and most of the parents have a middle or higher education. 69.1% of adolescents brushed their teeth once a day, most of them consumed a lot of sugary foods, and only 14.2% of them had dental appointment in the past 1 year. Most of the adolescents believed that their oral health was good, 34% of the adolescents had dental caries, and most of the adolescents had dental calculus (86.4%) and gingival bleeding (71.2%) (see Table 1).

**Table 1:** Characteristics of all subjects.

**Variables**		**N**	**%**
Province	Gansu	1,666	21.8
	Ningxia	2,987	39.1
	Shaanxi	2,995	39.2
Sex	Male	3,775	49.4
	Female	3,873	50.6
Age	12 years old	1,667	21.8
	13years old	1,922	25.1
	14years old	2,095	27.4
	15 years old	1,964	25.7
Household registration type	Urban	1,167	15.3
	Rural	6,481	84.7
Paternal education	Low	2,086	27.3
	Moderate	4,654	60.9
	High	322	4.2
	Unclear	586	7.7
Maternal education	Low	3,260	42.6
	Moderate	3,508	45.9
	High	239	3.1
	Unclear	641	8.4
Daily brushing frequency	Twice or more	1,147	15.0
	Once a day	5,281	69.1
	Not every day	419	5.5
	Occasionally or not	801	10.4
Sugar intake frequency	Low	991	13.0
	Moderate	4,593	60.0
	High	2,064	27.0
Dental appointments in the past 1 year	No	6,562	85.8
	Yes	1,086	14.2
Self-assessment of general health	Poor	307	4.0
	Moderate	2,689	35.2
	Good	4,651	60.8
Self-assessment of oral health	Poor	850	11.1
	Moderate	4,059	53.1
	Good	2,739	35.8
DMFT index	=0	5,050	66.0
	>0	2,598	34.0
The number of bleeding gums	=0	2,200	28.8
	>0	5,448	71.2
The number of teeth calculus	=0	1,042	13.0
	>0	6,606	86.4
Dental trauma	Yes	1,650	21.0
	No	5,998	79.0
Total		7,648	100

The average total score of Child-OIDP was 21.94 ± 21.084. The Child-OIDP score of teenagers in Shaanxi Province were 19.24 ± 19.57, in Gansu Province were 24.58 ± 20.31, Ningxia Province were 23.18 ± 22.63. The prevalence rates of OHRQoL in adolescents in Shaanxi Province, Gansu Province and Ningxia Hui Autonomous Region were 80.4%, 92.1% and 82.6%, respectively. The difference was statistically significant (*P* < 0.001) (see Table 2). 83.8% of adolescents reported that the past oral problems had an impact on their daily lives. The highest reported impact was eating (65.3%), followed by brushing or gargling (46.8%), while the lowest impact was schooling (25.3%) (see Table 3).

**Table 2:** Prevalence of OHRQoL and Child-OIDP score of adolescents in different region of Northwest China.

		**N (%)**	**OHRQoL(n)**	**OHRQoL prevalence (%)**	***P* value**	**Child-OIDP score**	***P* value**
Province	Shaanxi	2,995 (39.2)	2,408	80.40	*P* < 0.001^*^	19.24 ± 19.57	*P* < 0.001^*^
	Gansu	1,666 (21.8)	1,534	92.10		24.58 ± 20.31	
	Ningxia	2,987 (39.1)	2,467	82.60		23.18 ± 22.63	

^*^P < 0.05 indicates a statistically significant difference.

**Table 3:** Prevalence of OHRQoL and distribution of Child-OIDP scores among adolescents in Northwest China.

**Variables**	**Prevalence (%)**	**Mean ±SD**	**N (%)**	**Slight (%)**	**Moderate (%)**	**Severe (%)**
Eating	65.3	1.08 ± 0.99	34.7	33.3	21.3	10.7
Pronouncing	31.6	0.46 ± 0.78	68.4	19.3	9.6	2.7
Brushing or gargling	46.8	0.77 ± 0.96	53.2	24.2	15.5	7.1
Schooling	25.3	0.40 ± 0.77	74.7	14.2	7.8	3.3
Sleeping	33.8	0.56 ± 0.90	66.2	17.6	10	6.2
Smiling	42.4	0.70 ± 0.95	57.6	22.4	12.7	7.3
Emotion	43.4	0.72 ± 0.96	56.6	22.6	13.1	7.7
Socializing	34.6	0.58 ± 0.91	65.5	17.4	11	6.2
Overall impact	83.8	21.94 ± 21.08	16.2	60.7	19.2	3.9

The factors influencing the prevalence of OHROoL include gender, household registration type and parental education level (see Table 4). The results showed that female (OR = 1.267, CI = 1.112–1.443), younger (OR = 0.725, CI = 0.602–0.874), rural (OR = 1.443, CI = 1.214–1.715), and teenagers with lower parental education (OR = 0.692, CI = 0.595–0.805) were more likely to occur OHRQoL. The prevalence of OHRQoL was higher in adolescents who had high frequency of sugar intake (OR = 1.720, CI = 1.407–2.104), saw a doctor within the past 1 year (OR = 1.294, CI = 1.064–1.574), poor self-assessed of general health and oral health (OR = 3.092, CI = 2.285–4.185), DMFT index>0 (OR = 1.190, CI = 1.037–1.366). The frequency of brushing daily had no significant difference in the prevalence of OHRQoL (*P* > 0.05) (see Table 5).

**Table 4:** Univariate analysis of independent variables and OHRQoL prevalence.

**Variables**		***N* (%)**	**OHRQoL (N)**	**OHRQoL prevalence (%)**	***P* value**
Sex	Male	3,775 (49.4)	3,126	82.80	0.02^*^
	Female	3,873 (50.6)	3,283	84.80	
Age	12 years old	1,667 (21.8)	1,429	85.70	0.009^*^
	13years old	1,922 (25.1)	1,632	84.90	
	14years old	2,095 (27.4)	1,732	82.70	
	15 years old	1,964 (25.7)	1,616	82.30	
Household registration type	Urban	1,167 (15.3)	897	76.90	<0.001^*^
	Rural	6,481 (84.7)	5,512	85.00	
Paternal education	Low	2,086 (27.3)	1,832	87.80	<0.001^*^
	Moderate	4,654 (60.9)	3,850	82.70	
	High	322 (4.2)	248	77.00	
	Unclear	586 (7.7)	479	81.70	
Maternal education	Low	3,260 (42.6)	2,860	87.70	<0.001^*^
	Moderate	3,508 (45.9)	2,848	81.20	
	High	239 (3.1)	181	75.70	
	Unclear	641 (8.4)	520	81.10	
Daily brushing frequency	Twice or more	1,147 (15.0)	923	80.50	0.004^*^
	Once a day	5,281 (69.1)	4,439	84.10	
	Not every day	419 (5.5)	362	86.40	
	Occasionally or not	801 (10.4)	685	85.50	
Sugar intake frequency	Low	991 (13.0)	775	78.20	<0.001^*^
	Moderate	4,593 (60.0)	3,866	84.20	
	High	2,064 (27.0)	1,768	85.70	
Dental appointments in the past 1 year	No	6,562 (85.8)	5,471	83.40	0.013^*^
	Yes	1,086 (14.2)	938	86.40	
Self-assessment of general health	Poor	307 (4.0)	283	92.20	<0.001^*^
	Moderate	2,689 (35.2)	2,313	86.00	
	Good	4,651 (60.8)	3,812	82.00	
Self-assessment of oral health	Poor	850 (11.1)	794	93.40	<0.001^*^
	Moderate	4,059 (53.1)	3,462	85.30	
	Good	2,739 (35.8)	2,153	78.60	
DMFT index	0	5,050 (66.0)	4,170	82.60	<0.001^*^
	>0	2,598 (34.0)	2,239	86.20	
The number of bleeding gums	0	2,200 (28.8)	1,836	83.50	0.603
	>0	5,448 (71.2)	4,573	83.90	
The number of teeth calculus	0	1,042 (13.0)	845	81.10	0.011^*^
	>0	6,606 (86.4)	5,564	84.20	
Dental trauma	Yes	1,517 (19.8)	1,374	90.60	<0.001^*^
	No	4,250 (55.6)	3,418	80.40	

^*^P < 0.05 indicates a statistically significant difference.

**Table 5:** Binary logistic regression model between independent variables and OHRQoL prevalence.

**Variables**		**Wald**	**Standard error**	***P* value**	**OR**	**95% CI**
Sex	Male					
	Female	12.721	0.066	<0.001^*^	1.267	1.112–1.443
Age	12 years old					
	13years old	1.131	0.097	0.288	0.902	0.746–1.091
	14years old	10.506	0.094	0.001^*^	0.738	0.614–0.887
	15 years old	11.463	0.095	0.001^*^	0.725	0.602–0.874
Household registration type	Urban					
	Rural	17.249	0.088	<0.001^*^	1.443	1.214–1.715
Paternal education	Low					
	Moderate	5.673	0.085	0.017^*^	0.817	0.691–0.965
	High	1.172	0.184	0.279	0.819	0.571–1.175
	Unclear	0.475	0.165	0.491	0.892	0.645–1.234
Maternal education	Low					
	Moderate	22.836	0.077	<0.001^*^	0.692	0.595–0.805
	High	4.673	0.199	0.031^*^	0.650	0.440–0.961
	Unclear	10.427	0.152	0.001^*^	0.612	0.455–0.825
Daily brushing frequency	Twice or more					
	Once a day	0.188	0.09	0.664	1.040	0.872–1.24
	Not every day	1.597	0.169	0.206	1.238	0.889–1.726
	Occasionally or not	0.012	0.137	0.911	1.015	0.777–1.328
Sugar intake frequency	Low					
	Moderate	22.163	0.089	<0.001^*^	1.524	1.279–1.816
	High	27.938	0.103	<0.001^*^	1.720	1.407–2.104
Dental appointments in the past 1 year	No					
	Yes	6.671	0.1	0.01^*^	1.294	1.064–1.574
Self-assessment of general health	Good					
	Moderate	0.658	0.074	0.417	1.062	0.918–1.228
	Poor	4.726	0.223	0.03^*^	1.624	1.049–2.514
Self-assessment of oral health	Good					
	Moderate	28.927	0.071	<0.001^*^	1.462	1.273–1.679
	Poor	53.435	0.154	<0.001^*^	3.092	2.285–4.185
DMFT index	=0					
	>0	6.105	0.07	0.013^*^	1.190	1.037–1.366
The number of teeth calculus	=0					
	>0	4.105	0.089	0.043^*^	1.198	1.006–1.427
Dental trauma	Yes					
	No	58.137	0.099	<0.001^*^	0.471	0.388–0.571
	Unclear	16.848	0.113	<0.001^*^	0.629	0.504–0.785

^*^P < 0.05 indicates a statistically significant difference.

## Discussion

This is the first time that Child-OIDP questionnaire has been used to assess the OHRQoL among 12–15 years old adolescents in northwest China, and to explore the effects of socio-demographic characteristics and oral health-related behaviors on OHRQoL. This study found that the prevalence of oral diseases was higher and OHRQoL was poor in northwest China. There is also a gap in OHRQoL of adolescents in different provinces of Northwest China. The OHRQoL of adolescents in Gansu Province was the worst among the three provinces. Gansu Province is the most geographically complex province in China.

Association between socioeconomic and oral health-related quality of life is complex. Our study found that girls had worse OHRQoL than boys. It may be due to the psychological sensitivity of girls, oral problems are more likely to cause their anxiety emotions, thus affecting their daily life. There are different conclusions about the relationship between gender and OHRQoL. Studies have found that women's daily living behaviors are more likely to be affected by oral problems than men's (18). Parental education is also an important factor affecting OHRQoL, and adolescents from highly educated families are less affected by oral problems in their daily lives (19). Highly educated parents are more concerned about their children's oral health, and adolescents have access to more oral health services, so that they have good oral health status. Family income has also been shown to correlate with adolescent OHRQoL (20).

The study also found that the daily lives of younger people were more likely to be affected by oral problems (21), which may be related to a lack of oral health knowledge, which is insist with other study (22). Oral problems are intuitive factors that affect the daily life of adolescents. Severe caries can have a negative impact on daily life (23–25), which is the most common oral disease in adolescences (26). Poor oral condition may cause discomfort, affect the oral chewing function, and thus have an impact on the growth and development of adolescents. At the same time, the most common symptom caused by oral diseases is toothache, which is easy to cause anxiety and irritability, affecting their daily life such as sleep, study and diet. Studies have suggested that OHRQoL in children is negatively correlated with the incidence and severity of caries, while OHRQoL in adolescents is only negatively correlated with the incidence of caries (27). This study also found that adolescents with caries experience had worse OHRQoL and greater impact on daily life, consistent with the conclusions of Sweta (28, 29).

This study investigated the effects of brushing, sugar intake and dental appointment in the past 1 year on OHRQoL in adolescents. High sugar intake was found to be a contributing factor to oral problems in adolescents, consistent with Mbawalla HS et al. Sugar is one of the most direct factors leading to caries in adolescents, and a higher intake of sugar foods will lead to an increased risk of caries in adolescents, directly affecting their oral health. Brushing is the most basic behavior to keep the mouth clean and prevent oral diseases, and has a direct correlation with oral health. Studies have found that adolescents who brush their teeth more than twice a day have better oral health and are less affected in their daily life (30). However, this study did not find a significant association between brushing and OHRQoL in adolescents. In this study, the dental appointment rate in the past 1 year was used as the utilization rate of adolescent oral health services, and it was found that adolescents with dental appointment experience in 1 year had higher prevalence of OHRQoL and Child-OIDP score and worse quality of daily life. However, Kassim (31) did not find an association between them.

Self-assessed of oral health is a common indicator of epidemiological investigation. Self-assessed of oral health can help doctors better understand the oral condition of patients, and can indirectly reflect the oral health knowledge, attitude and behavior of patients. OHRQoL is influenced by self-assessed oral health. This study found that adolescents with poor self-assessed of oral health were more likely to be affected by oral problems (32). In investigating the residents in eastern and western regions obtained the consistent conclusion (33). Worse oral health is associated with worse self-assessed of oral health status and OHRQoL (34).

## Conclusion

This study found that the oral health-related daily life of adolescents in Northwest China was greatly affected by oral health-related behaviors, eating and brushing teeth or gargling were the most affected. Adolescents who ate more sugary foods and had dental appointment in past 1 year were more likely to have oral problems in their daily lives. We should strengthen oral health education for adolescents, help them to establish a positive attitude toward oral health, develop good habits, promote oral health and improve the quality of life. Policy makers should pay more attention to the dissemination of oral health knowledge in schools and promote the availability of oral health service resources.

## Limitation

There are still some limitations in this study. Firstly, due to the availability of data, only three of the five Northwest regions were included in this study. Tibet Autonomous Region which contains the largest number of ethnic minorities and Qinghai Province were not included, and there was a slight regional bias in the samples. Second, our study is a cross-sectional study, it is not possible to infer a causal relationship between independent variables and the prevalence and score of OHRQoL. Future prospective studies should be conducted to analyze the causal relationship.

## Data Availability

The raw data supporting the conclusions of this article will be made available by the authors, without undue reservation.
